# Supplementary material for: Osteoarthritis and Diabetes: Where Are We and Where Should We Go?
Source: Diagnostics (Basel). 2023 Apr 10;13(8):1386. doi: 10.3390/diagnostics13081386 (PMC10137010; doi:10.3390/diagnostics13081386)
Supplement: Supplementary file 1 [file diagnostics-13-01386-s001.zip › diagnostics-2227016-supplementary.pdf]

diagnostics

MDPI

Supplementary Material

Table S1. PubMed.

Search	String	Items Found
#1	"diabetes mellitus" OR diabetes	48,737
#2	(osteoarthritis) OR osteoarthrosis OR arthritis OR "degenerative arthritis" OR "joint pain"	19,667
#3	(epidemiology OR incidence OR progression)	232,921
#4	(medication OR "hypoglycemic agents" OR "antidiabetic agents")	337,296
#5	#1 AND #2 AND #3 AND #4<br>((("diabetes mellitus" OR diabetes AND ((clinicaltrial[Filter] OR meta-analysis[Filter] OR randomizedcontrolledtrial[Filter] OR review[Filter] OR systematicreview[Filter]) AND (fft[Filter]) AND (humans[Filter]) AND (english[Filter]) AND (alladult[Filter]))) AND ((osteoarthritis) OR osteoarthrosis OR arthritis OR "degenerative arthritis" OR "joint pain" AND ((clinicaltrial[Filter] OR meta-analysis[Filter] OR randomizedcontrolledtrial[Filter] OR review[Filter] OR systematicreview[Filter]) AND (fft[Filter]) AND (humans[Filter]) AND (english[Filter]) AND (alladult[Filter])))) AND ((epidemiology OR incidence OR progression) AND ((clinicaltrial[Filter] OR meta-analysis[Filter] OR randomizedcontrolledtrial[Filter] OR review[Filter] OR systematicreview[Filter]) AND (fft[Filter]) AND (humans[Filter]) AND (english[Filter]) AND (alladult[Filter])))) AND ((medication OR "hypoglycemic agents" OR "antidiabetic agents") AND ((clinicaltrial[Filter] OR meta-analysis[Filter] OR randomizedcontrolledtrial[Filter] OR review[Filter] OR systematicreview[Filter]) AND (fft[Filter]) AND (humans[Filter]) AND (english[Filter]) AND (alladult[Filter]))) Filters: Full text, Clinical Trial, Meta-Analysis, Randomized Controlled Trial, Review, Systematic Review, Humans, English, Adult: 19+ years, from 1972 - 2022	172

Table S2. Scopus.

Search	String	Items Found
#1	ALL ("diabetes mellitus" OR diabetes)	2,639,261
#2	ALL (osteoarthritis OR osteoarthrosis OR arthritis OR "degenerative arthritis" OR "joint pain")	1,425,024
#3	ALL ((epidemiology OR "incidence" [mesh AND terms] OR incidence OR progression))	34,539
#4	ALL ((medication OR "hypoglycemic agents" OR "hypoglycemic agents" [mesh AND terms] OR "antidiabetic agents"))	2,543
#5	#1 AND #2 AND #3 AND #4<br>(((ALL ("diabetes mellitus" OR diabetes) AND (ALL (osteoarthritis OR osteoarthrosis OR arthritis OR "degenerative arthritis" OR "joint pain")) AND (ALL ((epidemiology OR "incidence" [mesh AND terms] OR incidence OR progression))) AND (ALL ((medication OR "hypoglycemic agents" OR "hypoglycemic agents" [mesh AND terms] OR "antidiabetic agents")))	106

Table S3. Web of Science.

Search	String	Items Found
#1	ALL=("diabetes mellitus"[MeSH Terms] OR diabetes)	1,145,335
#2	ALL=(osteoarthritis OR osteoarthrosis OR arthritis OR "degenerative arthritis" OR "joint pain")	560,979
#3	ALL=(epidemiology OR "incidence" [mesh AND terms] OR incidence OR progression)	2,170,436
#4	ALL=(((medication OR "hypoglycemic agents" OR "hypoglycemic agents" [mesh AND terms] OR "antidiabetic agents")))	392,512
#5	#1 AND #2 AND #3 AND #4<br>ALL=("diabetes mellitus"[MeSH Terms] OR diabetes) AND ALL=(osteoarthritis OR osteoarthrosis OR arthritis OR "degenerative arthritis" OR "joint pain") AND ALL=(epidemiology OR "incidence" [mesh AND terms] OR incidence OR progression) AND ALL=(((medication OR "hypoglycemic agents" OR "hypoglycemic agents" [mesh AND terms] OR "antidiabetic agents")))	232

Table S4. Cochrane library.

Search	String	Items Found
#1	("diabetes mellitus" OR diabetes)	102423
#2	(osteoarthritis OR osteoarthrosis OR arthritis OR "degenerative arthritis" OR "joint pain")	49208
#3	(epidemiology OR incidence OR progression)	269396
#4	(medication OR "hypoglycemic agents" OR "antidiabetic agents")	101292
#5	#1 AND #2 AND #3 AND #4<br>("diabetes mellitus" OR diabetes) AND (osteoarthritis OR osteoarthrosis OR arthritis OR "degenerative arthritis" OR "joint pain") AND (epidemiology OR incidence OR progression) AND (medication OR "hypoglycemic agents" OR "antidiabetic agents")	237
